# Participant-reported personal utility of genetic testing for Parkinson’s disease and interest in clinical trial participation

**DOI:** 10.1038/s41531-024-00805-z

**Published:** 2024-10-25

**Authors:** Hannah Oas, Lola Cook, Tae-Hwi Schwantes-An, Laurence E. Walsh, Anne-Marie Wills, Ignacio F. Mata, Martha A. Nance, James C. Beck, Anna Naito, Karen Marder, Roy N. Alcalay, Jennifer Verbrugge

**Affiliations:** 1 Department of Medical and Molecular Genetics, Indiana University School of Medicine, Indianapolis, IN USA; 2 Department of Neurology and Pediatrics, Indiana University School of Medicine, Indianapolis, IN USA; 3 Department of Neurology, Massachusetts General Hospital, Boston, MA USA; 4 Department of Genomic Medicine, Lerner Research Institute, Cleveland Clinic, Cleveland, OH USA; 5 Struthers Parkinson’s Center, Health Partners Institute, Minneapolis, MN USA; 6 Parkinson’s Foundation, New York, NY USA; 7 Department of Neurology, Columbia University Irving Medical Center, New York, NY USA; 8 Neurological Institution, Tel Aviv Sourasky Medical Center, Tel Aviv, Israel; 9 Tel Aviv University, Faculty of Medicine and Sagol School of Neuroscience, Tel Aviv, Israel

**Keywords:** Parkinson's disease, Parkinson's disease, Parkinson's disease, Outcomes research, Risk factors

## Abstract

Genetic testing for Parkinson’s disease (PD) is infrequently performed due to perceptions of low utility. We investigated the personal utility in PD GENEration and how results lead to enrollment in additional research studies. Participants (*n* = 972) underwent genetic testing, results disclosure, genetic counseling, and completed a survey examining the perceived personal utility of their results and interest in participating in additional studies. Most participants found their genetic test results useful, including satisfying curiosity (81%), feeling good about helping the medical community (80%), and having information to share with family (77%). There were no significant differences in responses based on result type. Forty-five percent of participants expressed interest in participating in research studies; whereas 16% of participants confirmed enrollment. Our results suggest that participants find personal utility in genetic testing regardless of results. Although participants may be interested in enrolling in additional research, they may need support and resources.

## Introduction

While most cases of Parkinson’s disease (PD) are sporadic, 15% of people with Parkinson’s (PwP) have a family history of PD and at least 5–10% of PwP carry a genetic variant with a predisposition to developing PD^1^. The most common genetic causes of PD include variants in the genes *GBA1, LRRK2*, and *PRKN*, although many other gene variants have been linked to PD^2,3^. However, genetic testing is not a standard practice in clinical care for PD. Most providers report ordering genetic testing infrequently due to concerns over the lack of clinical utility and poor insurance coverage^4^. Clinical utility describes an intervention’s impact on diagnosis, treatment, or management^5^. Currently, the diagnosis of PD is made based on clinical presentation and disease management is unlikely to change based on the results of genetic testing^6,7^. However, positive genetic test results can permit enrollment in gene-specific clinical trials that would be otherwise unavailable—one potential clinical benefit of genetic testing for PD. Similarly, gene-specific clinical trials rely on PwP receiving genetic testing to identify appropriate participants.

In addition, providers may believe that patients are not interested in receiving genetic testing for PD due to their perception of its limited clinical utility^4^. Yet, PwP may find personal utility in their test results even without clinical impact. This disconnect between provider perceptions and patient interest is suggested by the large numbers of individuals who have pursued genetic testing for Parkinson’s disease through direct-to-consumer companies on their own^4^. Personal utility describes the non-medical impacts of an intervention. Examples of personal utility provided by genetic testing include information for reproductive planning, mental preparation for the future, increased self-knowledge, and the opportunity to share information with relatives^8,9^. Prior studies demonstrate that PwP and their family members are interested in genetic testing for use in medical decision-making, financial planning, and informing family members’ risks^10,11^. There is a vast need to better understand the value of genetic testing for people with PD from the patient’s perspective; however, there are very few studies surveying PwP who have completed genetic testing to assess its impact on patients^12^.

In this study, we aimed to assess the impact of genetic testing on the lives of people with PD. We hypothesized that PwP would find personal utility in their genetic test results, regardless of the positive or negative result type. We sought to identify the elements of personal utility that were most useful to PwP who received genetic test results in a research setting, as well as the medical and life changes experienced by participants due to their genetic test results. We hypothesized that identifying individuals with a genetic form of PD would encourage enrollment in gene-specific clinical trials.

## Results

### Sample characteristics

Among 1622 participants who were sent the impact survey, we received 972 responses (60% response rate). The demographic and clinical characteristics of responders versus nonresponders were not significantly different (all *P* values > 2.5 × 10^−4^) (Table 1). Forty-three percent (421/972) of participants identified as female and the average age of the cohort was 64.7 years (SD 9.6 years). The cohort was largely White and highly educated, with 96% (924/963) of participants identifying as White and 95% (927/972) having at least some college education. Demographic data is summarized in Table 1. Among the responders, 14% (137/972) had positive genetic testing results. Variants were identified in all genes on the genetic testing panel, except for *VPS35*. *GBA1* variants were most commonly identified, with 8% (78/972) of all participants having a variant in this gene. A breakdown of genetic testing results by gene can be found in Table 2 in the supplement.

**Table 1:** Demographic and clinical characteristics of responders and non-responders

	Total participants (*n* = 1622)	Participants who did not respond (*n* = 650)	Participants who did respond (*n* = 972)	*P* value^a^
Mean age in years (SD)	64.7 (10.1)	64.8 (10.7)	64.7 (9.6)	0.599
Mean age of PD onset in years (SD)	59.2 (10.7)	59.0 (11.1)	59.4 (10.4)	0.471
Early-onset (<50 years) PD (%)	290/1622 (18%)	129/650 (20%)	161/972 (17%)	0.098
Positive test (%)	241/1622 (15%)	104/650 (16%)	137/972 (14%)	0.319
First-degree relative with PD (%)	352/1372 (26%)	130/548 (24%)	222/824 (27%)	0.186
Mean MoCA score (SD)	26.5 (2.9)	26.2 (3.2)	26.7 (2.7)	0.006
Gender (%)				1.000
Female	702/1622 (43%)	281/650 (43%)	421/972 (43%)	
Race (%)				0.028
African American	16/1600 (1%)	8/637 (1%)	8/963 (1%)	
Asian & Pacific Islanders	34/1600 (2%)	16/637 (3%)	18/963 (2%)	
White	1516/1600 (95%)	592/637 (93%)	924/963 (96%)	
Indigenous	2/1600 (<1%)	2/637 (<1%)	0/963 (0%)	
Other	32/1600 (2%)	19/637 (3%)	13/963 (1%)	
Ethnicity (%)				0.170
Hispanic	84/1622 (5%)	40/650 (6%)	44/972 (5%)	
High-risk ancestry^b^ (%)	299/1622 (18%)	135/650 (21%)	164/972 (17%)	0.050
Education (%)				0.002
High school degree or fewer years	95/1622 (6%)	50/650 (8%)	45/972 (5%)	
Bachelors degree or some college	796/1622 (49%)	337/650 (52%)	459/972 (47%)	
Graduate degree	731/1622 (45%)	263/650 (40%)	468/972 (48%)	
Marital Status (%)				0.717
Single or never married	141/1621 (9%)	52/649 (8%)	89/972 (9%)	
Married/domestic partnership	1280/1621 (79%)	515/649 (79%)	765/972 (79%)	
Widowed, divorced, or separated	200/1621 (12%)	82/649 (13%)	118/972 (12%)	
Employment Status (%)				0.626
Employed	526/1622 (32%)	206/650 (32%)	320/972 (33%)	
Not employed	1096/1622 (68%)	444/650 (68%)	652/972 (67%)	
Living Situation (%)				0.181
Own home or with family	1595/1622 (98%)	636/650 (98%)	959/972 (99%)	
Assisted living facility or nursing home	11/1622 (1%)	4/650 (1%)	7/972 (1%)	
Other	16/1622 (1%)	10/650 (2%)	6/972 (1%)	

*MoCA* montreal cognitive assessment, *PD* Parkinson’s disease, *SD* standard deviation.

^a^No comparisons statistically significant after multiple testing correction (*P* > 2.5 × 10^−4^).

^b^High-risk ancestry includes Ashkenazi Jewish, Spanish Basque, and North African Berber.

**Table 2:** Personal utility by genetic testing result

Survey item	% Total endorsed as useful (*n*/*n*_responses_)	% Gene-positive endorsed as useful (*n*/*n*_responses_)	% Gene-negative endorsed as useful (*n*/*n*_responses_)	*P* value^a^
Satisfy my curiosity	774/957 (81%)	115/135 (85%)	659/822 (80%)	0.209
Feel good about helping the medical community	770/957 (80%)	112/137 (82%)	658/820 (80%)	0.768
Simply to provide information	747/955 (78%)	116/137 (85%)	631/818 (77%)	0.062
Feel good about having information for my family members	734/958 (77%)	107/137 (78%)	627/821 (76%)	0.738
Contribute to my self-knowledge	671/959 (70%)	113/137 (82%)	558/822 (68%)	0.001
Feel good about taking responsibility for my children’s health	601/952 (63%)	84/136 (62%)	517/816 (63%)	0.794
Help to better understand my health	493/959 (51%)	85/137 (62%)	408/822 (50%)	0.009
Help me cope with my health risks	468/958 (49%)	76/136 (56%)	392/822 (48%)	0.093
Help me feel more in control of my health	467/958 (49%)	71/136 (52%)	396/822 (48%)	0.436
Improve communication with my family members	455/957 (48%)	70/137 (51%)	385/820 (47%)	0.420
Help me feel more in control of my life	452/956 (47%)	73/136 (54%)	379/820 (46%)	0.128
Help me to use social programs, like resources and services	382/957 (40%)	63/137 (46%)	319/820 (39%)	0.141
Help with life planning	377/958 (39%)	64/137 (47%)	313/821 (38%)	0.070
Help me or my family mentally prepare for the future	358/960 (37%)	60/137 (44%)	298/823 (36%)	0.109
Inform my plans for school or career	202/957 (21%)	34/136 (25%)	168/821 (20%)	0.277
Inform my decisions about having children	168/946 (18%)	29/135 (21%)	139/811 (17%)	0.271

^a^*P*-value refers to comparisons between gene-negative and gene-positive participants. No comparisons statistically significant after multiple testing correction (*P* > 2.5 × 10^−4^).

### Personal utility of genetic testing

Participants were provided with 16 examples of ways their genetic test results could be personally useful (Table 2). Those who responded that an item was “somewhat useful”, “useful”, or “extremely useful”, were collapsed into the “useful” category. Participants reported the following aspects of personal utility to be most useful: satisfying their curiosity (81%, 774/957), feeling good about helping the medical community (80%, 770/957), simply providing information (78%, 747/955), feeling good about having information for my family members (77%, 734/958), and contributing to their self-knowledge (70%, 671/959). Participants reported the following aspects of personal utility to be the least useful: using social programs like resources and services (40%, 382/957), helping with life planning (39%, 377/958), helping mentally plan for the future (37%, 358/960), informing plans for school or career (21%, 202/957), and informing decisions about having children (18%, 168/946). There were no significant differences in personal utility between participants with positive genetic testing and participants with negative genetic testing (all *P* values > 2.5 × 10^−4^), although a larger proportion of participants with positive results endorsed nearly all items as personally useful (Table 2). Participant responses to the personal utility items that are grouped by type of positive results are shown in the supplement (Supplementary Table 5). There were no significant differences (all *P* values > 2.5 × 10^−4^) identified in aspects of personal utility that were endorsed by patients segregated by sex, ethnicity, Montreal Cognitive Assessment (MoCA) score, duration of PD, high-risk ancestry, family history of PD, marital status, employment status, or years of education (Supplementary data).

Significant differences in the age of participants who found personal utility in genetic testing were identified (Supplementary Table 3). Participants who found genetic testing to be useful for informing plans for school or career (mean [SD] age of 61.2 [10.6] vs. 66.0 [9.1], *P* = 2.3 × 10^−8^), informing decisions about having children (mean [SD] age of 60.6 [11.2] vs. 65.8 [9.0], *P* = 6.8 × 10^−8^), and helping them cope with health risks (mean [SD] age of 63.3 [10.0] vs. 66.0 [8.9], *P* = 0.0001) were significantly younger than participants who found their genetic testing results not useful for the same purposes.

### Medical and life changes after genetic testing

Participants were asked about changes that had occurred in their personal lives, such as a job change or change in relationship status, and changes to their medical care since receiving their genetic testing results (Table 3). Of participants who reported a medical change, most did not believe this change was related to their genetic test results. Of the 162 participants who had started a new medication, seven believed this was related to genetic test results. Of the 18 participants who had a PD-related surgery, two believed this was associated with their genetic test results. We did not collect additional information about the type of medication or surgery. Similarly, most participants who reported a life change like a job change, relationship change, or moving to a new residence did not believe this change was related to their genetic test results.

**Table 3:** Participant-reported medical and life changes and perceived relationship to genetic test results

Survey item	Total (*n* = 972)		Gene-positive (*n* = 137)		Gene-negative (*n* = 835)	
	*n* (%) reported this has happened, and it is related to gene test results.	*n* (%) reported this has happened, but it is not related to gene test results.	*n* (%) reported this has happened, and it is related to gene test results.	*n* (%) reported this has happened, but it is not related to gene test results.	*n* (%) reported this has happened, and it is related to gene test results.	*n* (%) reported this has happened, but it is not related to gene test results.
I have become interested in enrolling in a Parkinson’s disease research study.	139 (14%)	299 (31%)	45 (33%)	36 (26%)	94 (11%)	263 (31%)
I have enrolled or I am in the process of enrolling in a Parkinson’s disease research study.	40 (4%)	120 (12%)	14 (10%)	9 (7%)	26 (3%)	111 (13%)
I have left a job.	3 (< 1%)	44 (5%)	2 (1%)	14 (10%)	1 (< 1%)	30 (4%)
I have lost a job.	2 (< 1%)	18 (2%)	0 (0%)	4 (3%)	2 (< 1%)	14 (2%)
I have started a new job.	0 (0%)	12 (1%)	0 (0%)	1 (< 1%)	0 (0%)	11 (1%)
I have had a change in a significant relationship (marriage, break-up, etc.).	1 (< 1%)	15 (2%)	0 (0%)	4 (3%)	1 (< 1%)	11 (1%)
I have moved to a new residence.	5 (< 1%)	29 (3%)	1 (< 1%)	5 (4%)	4 (< 1%)	24 (3%)
I have started a new medication.	7 (< 1%)	155 (16%)	3 (2%)	24 (18%)	4 (< 1%)	131 (16%)
I have had surgery related to Parkinson’s.	2 (< 1%)	16 (2%)	0 (0%)	1 (< 1%)	2 (< 1%)	15 (2%)
I discussed advanced care planning or advanced directives with my physician and/or family.	18 (2%)	94 (10%)	7 (5%)	11 (8%)	11 (1%)	83 (10%)

Twenty-eight percent (273/972) of participants reported sharing their genetic test results with a healthcare provider, including 31% (43/137) of participants with positive results.

Participants had the option of writing in additional medical or life changes they experienced since receiving genetic testing results. Twenty-three percent (219/972) of participants responded to the open-text question. Fifty-seven percent (124/219) of responses were excluded from analysis due to participants expressing that no medical or life changes had occurred or due to unrelated comments. Thirty percent (65/219) of the open text responses reported medical changes, such as a change in medication, an injury or COVID-19 infection. The remaining 13% (30/219) of responses were sorted into categories based on similar themes. The categories include lifestyle changes (16 responses), positive emotions (8 responses), informed family members (4 responses), and negative emotions (2 responses). Examples of responses from each category are included in Supplementary Table 4.

### Clinical trial interest and enrollment

**Fig. 1:**
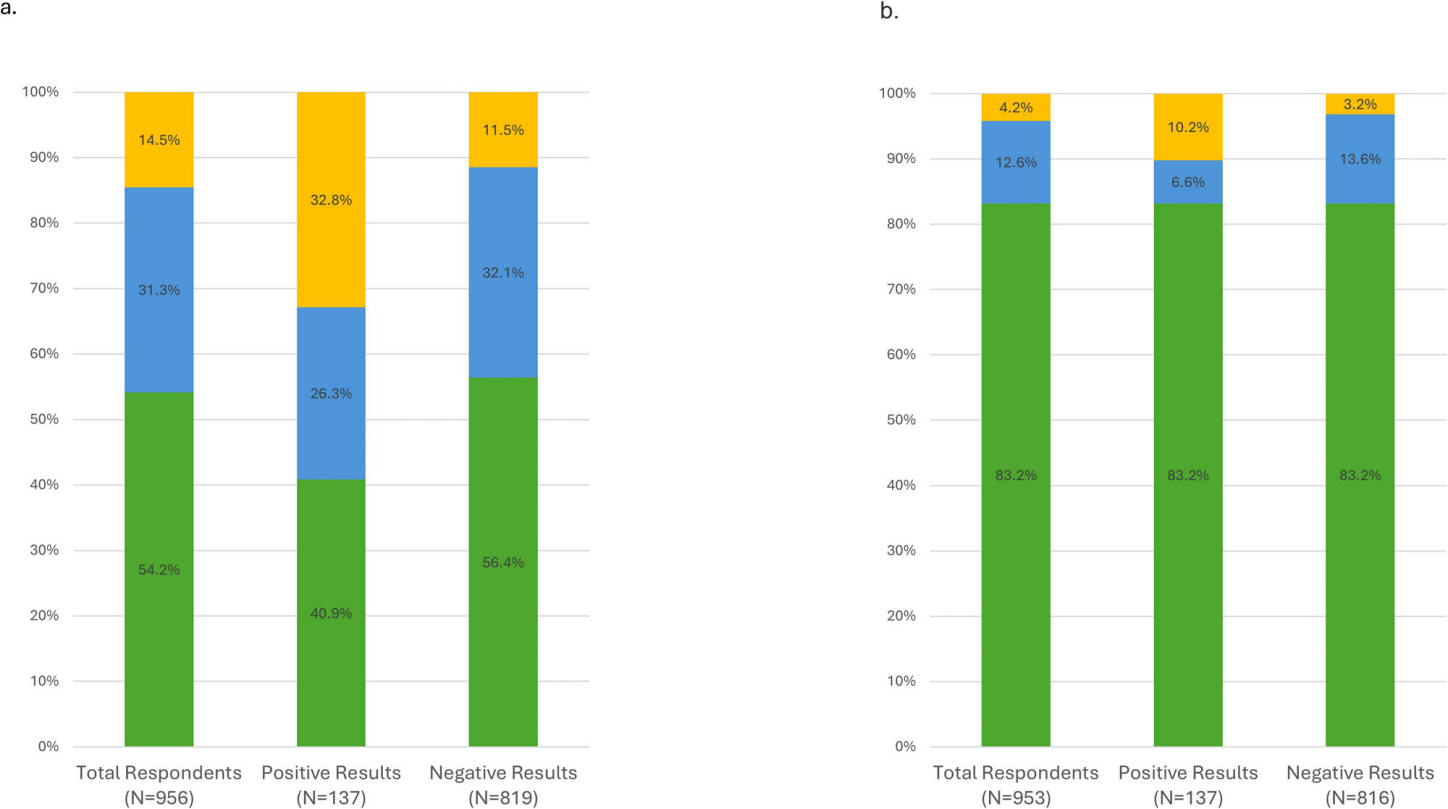
Clinical trial interest and enrollment. Participant-reported (**a**) interest and (**b**) enrollment in Parkinson’s disease research studies among all respondents, respondents with positive results, and respondents with negative results. Yellow bars indicate participants with interest or enrollment due to gene test results. Blue bars indicate participants with interest or enrollment that were not due to gene test results. Green bars indicate participants who have not become interested or enrolled in PD research since receiving gene test results.

Participants were asked about their interest in participating in further PD research studies (Fig. 1). Forty-five percent (438/972) of participants indicated that they were interested in participating in a PD research study. Fifty-nine percent (81/137) of participants with positive genetic testing results indicated they were interested in participating in a PD research study, compared to 44% (357/819) of participants with negative genetic testing results (*P* = 8.3 × 10^−4^). Enrollment in a research study was less common. Sixteen percent (160/972) of total study participants reported enrolling in a research study or initiating the enrollment process since receiving genetic test results, including 17% (23/137) of participants with positive results and 16% (137/835) of participants with negative results.

Not all participants attributed their interest in further clinical trial participation to receiving their genetic test results. Of the 81 gene-positive participants who were interested in further clinical trial participation, 56% (45/81) reported interest occurred due to their genetic test results while 44% (36/81) of participants believed their interest in participating in clinical trials was unrelated to genetic test results. Among the 357 gene-negative participants who were interested in further clinical trial participation, 26% (94/357) attributed their interest to the results of their genetic test while 74% (263/357) did not (Table 3). Interest and enrollment in additional research due to genetic results among genetic subgroups are shown in the supplement (Supplementary Table 6).

Participants had the option of writing in the name or principal investigator of a research study they had enrolled in. Of 114 responses, two indicated enrollment in gene-specific research studies for *GBA1* variant carriers. A third participant indicated interest in a clinical trial for *GBA1* variant carriers but had not enrolled. Other drug intervention enrollments included: Trial of Parkinson’s and Zoledronic Acid (NCT03924414, 7 responses), alpha-synuclein targeted trials (4 responses), and glucagon-like peptide-1 receptor agonist (GLP-1) studies (4 responses), among others. The remainder reported enrollment in other non-drug treatment interventions or observational studies. Several noted participation in studies supported by various Parkinson’s disease foundations including Fox Insight (12 responses) and Parkinson’s Progression Markers Initiative (NCT05065060, 8 responses).

## Discussion

Genetic testing for PD is more accessible than ever before through research studies like PD GENE, direct-to-consumer testing, and traditional clinical settings with a physician or genetic counselor^13,14^. However, there is the potential for discrepancy between perceived utility by providers and what PwP may actually experience with such testing, which has not been explored in depth by researchers. This study aimed to address this gap in the literature by investigating the personal utility reported by PwP who underwent genetic testing for PD in the context of a research study. In addition, we investigated the impact of genetic testing on life changes and enrollment in additional research studies, including gene-specific clinical trials. Importantly, 80% of participants found some personal utility in their genetic test results, regardless of whether the results were positive or negative. In addition, 45% of participants were interested in participating in additional research studies, and 16% of participants actually enrolled in another study.

Most participants found genetic testing for PD to be personally useful in multiple ways. Greater than 75% of participants reported that their genetic test results were useful for satisfying their curiosity, making them feel good about helping the medical community, providing information, and having information to share with family members. There were no significant differences between the proportion of PwP with positive results and PwP with negative results who found their genetic testing results useful for any aspect of personal utility. When evaluating responses by positive results broken down by genetic subgroup, we observed that each group appeared to endorse (as most useful) the same top four personal utility items as noted above, although we point out that these subsets by variant type were small, limiting interpretation (for details refer to Supplementary, Table 5). Multiple prior studies have demonstrated that PwP and their family members are interested in genetic testing for many reasons, including medical management, informing family members risks, or financial planning^10,11^. Our study adds to this knowledge by demonstrating that most people who receive genetic testing for PD do find personal utility in their test results, even when clinical utility is limited. Participants who found their genetic test results to be useful for informing plans for school or career, informing decisions about having children, and helping them cope with their health risks were significantly younger on average than participants who reported that genetic testing was not personally useful. This data suggests that some elements of personal utility provided by genetic testing, such as reproductive decision-making and career planning, may be more valuable for younger individuals.

Most participants with negative genetic test results report finding genetic testing useful in multiple ways, despite not receiving an explanation for why they developed PD. Prior research reported that people with negative genetic test results find comfort in their results while still understanding that other factors can contribute to disease risk^15^. This is supported by the open-text responses provided by some gene-negative participants who reported feeling relieved after receiving their negative genetic test results. Additionally, 80% of participants with negative results endorsed feeling good about helping the medical community by participating in the study, an example of participant-reported research altruism. Prior research has found altruism to be a common motivation for participation in research, particularly in combination with a perceived benefit to participation such as learning health information^16,17^.

One of the aims of our study was to assess the clinical trial interest and enrollment behavior of participants after genetic testing. Overall, less than half of the participants expressed interest in additional research studies in general, which is less than what would be expected based on prior reports^18–20^. This could be attributed to the timing of the survey that was administered during the COVID pandemic. In addition, there was less interest in genetic versus nongenetic research studies as previously reported^18,21^. We hypothesized that identifying PwP with positive genetic results would lead to enrollment in gene-specific clinical trials. However, only one-third of participants with positive results reported interest in participating in a PD research study due to their positive genetic test and, less than a third of those participants had enrolled in another PD study. We did not perform statistical analyses of responses by genetic variant type because subsets were too small to allow for meaningful interpretation, although we have provided the data in the supplement (Supplementary Table 6). Participants were not required to write in the name of a clinical trial they had enrolled in or were interested in, but of those who did, three participants mentioned gene-specific clinical trials. We are aware of at least six gene-specific clinical trials for *LRRK2* and *GBA1* in the United States that had overlapping enrollment periods to this study, including observational and interventional trials (NCT03545425, NCT04668898, NCT04101968, NCT03234478, NCT02906020, NCT04127578). However, at the time of recruitment, there were no phase 2 clinical trials for either *LRRK2* or *GBA1*. Thus, we observed that those with positive results did not necessarily participate in research studies or enroll based on their genetic status.

There are many reasons why participants may not enroll in additional research studies including those related to a positive result. There may be accessibility issues, a perceived burden, risk aversion to interventions that may cause side effects, the possibility of getting a placebo, or not meeting study inclusion criteria^20,22^. At the time of survey completion, only 30% of participants had shared their genetic test results with a provider. Conversations with a healthcare provider about additional research participation perhaps had not taken place for participants who may have later enrolled in additional research after completing the survey. We highlight that the lack of large clinical trials for PD-related genes at the time could have played a role in trial enrollment. Additionally, enrolling participants with positive genetic results in gene-specific trials presents a challenge. The ideal trial participant may be a recently diagnosed individual who may not be compelled to enroll at the time because they perceive their symptoms to be manageable or are still processing receiving a new diagnosis^23^. For gene-specific clinical trials to enroll enough participants, PwP with genetic forms of PD will need to be identified through genetic testing; however, an important takeaway from this study is that simply identifying those with a genetic variant linked to PD may not be enough to lead to clinical trial enrollment for various reasons. There is a suggestion that it may matter who facilitates recruitment and how^13,18^; in the pilot portion of the PD GENE study participants endorsed the notion that they felt more like a “partner in care” when receiving results from a local site^13^. In addition to leveraging local multi-sites and community providers, increased engagement and targeted efforts will likely be necessary to improve recruitment to trials, especially in underrepresented populations^18,20^.

Clinical trials are rapidly evolving, with new trials emerging frequently such that it is not realistic to expect clinicians to be familiar with every clinical trial that could be an option for every patient. However, providers are an important point of contact for patients as research has shown that most clinical trial participants discuss enrollment with their healthcare provider^24^. A 2020 survey of physicians and nurses found that while over 80% of physicians feel comfortable providing information and discussing clinical trials with their patients, time constraints in appointments, difficulty accessing information about clinical trials, and a lack of time to evaluate clinical trials serve as major barriers to referral^24^. Less research is available about the behavior of genetic counselors when discussing clinical trials with their patients; however, providing education about research is highlighted as a critical role of genetic counselors in the National Society of Genetic Counselors’ most recent definition of genetic counseling^25^. Informing patients of relevant clinical trials is clearly within the scope of practice of both physicians and genetic counselors. Our study suggests that patients are interested in participating in research but may need to be encouraged to share test results with their healthcare providers and may need guidance in the next steps, such as assistance with finding and enrolling in studies. There are useful resources that can be shared with patients considering participation in clinical trials, including study search tools like ClinicalTrials.gov (www.clinicaltrials.gov) and the Fox Trial Finder (www.michaeljfox.org/trial-finder) and patient-facing materials available on foundation websites (www.parkinson.org/library/fact-sheets/research; www.michaeljfox.org/your-role-research. A detailed patient-facing digital guide to clinical trials is also available: https://www.michaeljfox.org/sites/default/files/media/document/PDEC_Patient_Guide_Digital_12.11.20_1.pdf). Of note, participants with both positive and negative results were interested in participating in PD research studies, highlighting the importance of assessing PwP’s interest in research regardless of test results.

Of those who had experienced change since receiving genetic test results, the most common was a medical event or medication adjustment. Life changes such as changes in relationship status or career changes were reported among both genetic test result groups as well as lifestyle changes including changes to diet and exercise habits. Two participants, both with positive test results, reported negative psychosocial reactions. One participant expressed concern over how the result would affect their family, while the other reported increased anxiety over having an additional disease. This is consistent with prior research demonstrating that although most PwP do not experience negative feelings after genetic testing, some individuals with positive results may have negative psychological reactions^26^. This finding highlights the importance of providing genetic counseling that includes anticipatory guidance to help patients prepare for and navigate complex emotions when they receive genetic test results.

Our study contains both strengths and limitations. A major strength of our study is the large sample size. However, our sample population is demographically homogenous with an overrepresentation of White and college-educated individuals. This could limit the generalizability of our findings to other populations. The study hopes to survey PwP living outside the U.S. as the PD GENE study expands, collaborating with other research groups including the Global Parkinson’s Genetics Program (GP2; https://gp2.org/about-gp2/) and the Latin American Research consortium on the genetics of Parkinson’s disease (LARGE-PD; https://large-pd.org/). Additionally, selection bias may have impacted our results as our participants must have some interest in research as they are already participants in the PD GENE study, which may have led us to an overestimate of the interest in research participation. The survey design with its structured data format could also limit ascertaining the breadth of participant experiences, which is likely to be heterogeneous; although, the addition of open text boxes allows for some free responses. Both the design of the study as well as specific survey questions precluded the ability to ascertain baseline, pre-test responses to some of the utility questions, which could impact interpretation of some results. Comparisons of personal utility items were not performed on genetic subgroups as counts were too small. Some participants received results from their own neurologist, which may have led to an underestimation of the participants who shared results with a healthcare provider; these participants may have responded they did not share results with their provider, perceiving it as unnecessary because their provider already knew their results. The timing of the survey may not have captured changes in behavior that occurred later due to testing including lifestyle changes, enrollment in additional research, or discussions about genetic test results with a healthcare provider after survey completion. Not sharing test results with health care providers could have skewed some of the clinical trial responses to the questions regarding enrollment, since physician input likely plays a role in decision-making. Lack of large clinical trials at the time of the study may also have played a role in enrollment numbers. Additionally, the COVID-19 pandemic may have had an effect on participants’ enrollment or interest in additional clinical trials. Importantly, most participants had negative genetic test results, which could have resulted in less interest in clinical trials and personal utility responses, although we observed that the result type did not always align with expected responses.

Future research could focus on the identification of modifiable factors that increase or reduce participants’ interest in future clinical trials. Standardization of trial information and referral processes among sites, which was not possible during this portion of the study, could improve recruitment into other research studies and should be explored. In addition, it will be important to investigate perceptions of personal utility among individuals with PD from other population groups. In the future, we plan to study the personal utility of genetic testing in PwP using qualitative methods. Reassessing clinical trial interest and enrollment will be important, as large gene-targeted trials such as ACTIVATE by Bial R&D (NCT05819359), LIGHTHOUSE, now LUMA, by Biogen (NCT05418673, NCT05348785), and PROPEL by Prevail Therapeutics (NCT04127578) and others continue to emerge.

In conclusion, we observed that most participants find personal utility in their PD genetic test results in multiple ways, including satisfying their curiosity, contributing to the “greater good” in medical research, and providing information to family members, with younger participants endorsing certain items more frequently (i.e. career planning, family planning). For most participants, genetic test results did not lead to medical or life changes. We observed that many research participants are interested in participating in other studies but may not enroll. This requires further exploration as to why PwP may not be enrolling in studies and suggests that physicians and genetic counselors should act as facilitators when there is expressed interest. While it may not be realistic for clinicians to be familiar with every emerging trial, clinical providers are an important point of contact to aid and provide resources for patients and their families who are interested in research.

## Methods

### Participants and process

PD GENEration (PD GENE) is an ongoing multicenter observational trial (NCT04057794, NCT04994015) providing complimentary genetic testing and genetic counseling to PwP. This study includes data from the pilot and clinical phases of the trial, which enrolled participants from September 2019 to November 2021 across multiple study sites nationwide and included approximately 2000 of the first participants enrolled in PD GENE. Outcome data from the pilot phase of the study determined the feasibility of recruitment, assessed participants’ knowledge and satisfaction, and evaluated the psychological impact of genetic testing and genetic counseling on PwP^13^. Participants were 18 years of age or older, met the Movement Disorder Society Clinical Diagnostic Criteria for probable PD based on clinical examination or chart review, were English-speaking, were able to provide informed consent and complete study activities, and were willing to undergo genetic testing and be informed of their results. Individuals with atypical parkinsonian disorders were excluded. Participants could self-refer to a study site via the Parkinson’s Foundation website or were recruited through a local study site.

After a baseline visit, all participants received genetic testing through a Clinical Laboratory Improvement Amendments (CLIA)-certified laboratory, Fulgent Genetics, by next-generation sequencing and duplication and deletion analysis of seven genes (*LRRK2*, *SNCA*, *VPS35*, *PRKN, PINK1*, *PARK7*, and *GBA1*) that have a well-established link to PD^2,3^. During the consenting process, additional pre-test education was provided to participants through a video that discussed the genetics of PD and the implications of genetic testing. Genetic testing results were disclosed to participants by a neurologist or genetic counselor via post-test genetic counseling locally, in person or via telemedicine, or remotely by a genetic counselor at a centralized site. The study was approved by a centralized and site institutional review board (IRB) and the Scientific Review and Executive Committees of the Parkinson’s Study Group.

Participants received genetic counseling between 3 and 12 weeks after their baseline visit. The impact survey was then sent to participants via e-mail or the postal service 90 days after the baseline visit, approximately 1–10 weeks after receiving disclosure of PD genetic test results and genetic counseling.

### Instrumentation

Details about the design of surveys have been previously described and the impact survey is included in the supplementary information^13^. The impact survey assessed several outcomes including: the psychological response to receiving genetic test results, how participants did or did not share their results, participants’ satisfaction with the method of genetic counseling service delivery, medical and life changes experienced by participants since receiving genetic test results, clinical trial interest and enrollment, and the personal utility of genetic test results. This study reports the final three outcomes.

Participant reported personal utility was assessed with a 16-item scale adapted from a Delphi study and a systematic literature review by Kohler et al.^8,9^. Participants selected options from a 5-response Likert scale ranging from “not at all useful” to “extremely useful”. The 16 items assessed 4 domains of personal utility including affective utility, cognitive utility, behavioral utility, and social utility as described in the systematic review by Kohler et al.^8^.

The Medical and Life Changes instrument included 10 novel items assessing potential medical, life and social changes that may have occurred since a participant completed genetic testing. Participants could report that the given change had not occurred, had occurred but the participant did not believe it was related to genetic testing results, or that the change had occurred and the participant believed it was related to genetic testing results. A free-response question with an open text box was available for participants to add any additional changes that had occurred since receiving genetic testing results.

### Statistical analysis

All statistical analyses and summary statistics were generated using R 4.2.3. Mean and standard deviations (SD) are provided for a summary of continuous variables. Categorical data are summarized by percentage and counts of each category over a total number of available participants. Differences in categorial variables were tested using Fisher’s exact test and continuous variables were tested using Mann–Whitney test to reduce false associations due to non-normality of distributions of tested variables. All tests were two-sided. After multiple test correction for 201 tests, the required *P* value threshold for statistical significance was 2.5 × 10^−4^.

## Supplementary information

Supplementary information

Supplementary Data-Participant-reported personal utility of genetic test results by demographic variables

## Data Availability

The datasets are available upon request from the corresponding author on reasonable request.
