# Supplementary material for: Participant-reported personal utility of genetic testing for Parkinson’s disease and interest in clinical trial participation
Source: NPJ Parkinsons Dis. 2024 Oct 25;10:202. doi: 10.1038/s41531-024-00805-z (PMC11511990; doi:10.1038/s41531-024-00805-z)
Supplement: Supplementary file 1 — Supplementary information [file 41531_2024_805_MOESM1_ESM.pdf]

Supplementary Information for Participant-reported personal utility of genetic testing for Parkinson’s disease and interest in clinical trial participation

PDGENE Impact Survey

Instructions: Thank you for your participation in this study. The purpose of this survey is to examine how genetic testing impacts people with Parkinson’s disease. Please answer the questions to the best of your ability.

1. Did you participate in genetic testing through our study PDGENE?
   - ☐ Yes
   - ☐ No
   - ☐ Do not remember

2. The following questions ask about how you have felt about receiving your genetic test results. Please indicate how much you had each specific feeling in the past week. Please select only one response per row.

	Not at all	A little	Somewhat	A good deal	A great deal
How ***upset*** did you feel about your genetic test result?	o	o	o	o	o
How ***happy*** did you feel about your genetic test result?	o	o	o	o	o
How ***anxious or nervous*** did you feel about your genetic test result?	o	o	o	o	o
How ***relieved*** did you feel about your genetic test result?	o	o	o	o	o
How ***sad*** did you feel about your genetic test result?	o	o	o	o	o
How ***frustrated*** did you feel about recommendations for your care based on the genetic test result?	o	o	o	o	o
How ***uncertain*** did you feel about what your genetic test result means for you?	o	o	o	o	o

How ***uncertain*** did you feel about what your genetic test result means for your child(ren) and/or family's risk of disease?	o	o	o	o	o
	**Not at all**	**A little**	**Somewhat**	**A good deal**	**A great deal**
How much did you feel that ***you understood clearly your choices for care*** based on the genetic test result?	o	o	o	o	o
How ***concerned*** did you feel that your genetic test result would affect your ability to get or keep health insurance?	o	o	o	o	o
How ***helpful*** was the information you received from your genetic test result in planning for the future?	o	o	o	o	o
How ***concerned*** did you feel that your genetic test result might make it hard for you to get a job or keep a job?	o	o	o	o	o

3. Have you shared your test results with others?
   - ☐ Yes
   - ☐ No

   A. If yes, whom have you told about your genetic test results? (check all that apply)
      - ☐ Spouse
      - ☐ Child (at least one of my children)
      - ☐ Parent (at least one parent)
      - ☐ Sibling (at least one sibling)
      - ☐ Extended family member (e.g. cousins)
      - ☐ A close friend
      - ☐ An acquaintance (e.g. work colleagues, neighbors)
      - ☐ A supervisor (e.g. boss, work supervisor).

☐ I have announced my results publicly (e.g. a holiday letter, Facebook or other social media posting, other media announcement).

☐ A healthcare provider (one or more). Select all that apply:

- ☐ Primary care doctor
- ☐ Parkinson's disease doctor other than the one who gave me my results
- ☐ Genetic counselor other than the one who gave me my results
- ☐ Mental health professional (psychiatrist, psychologist, social worker, etc)
- ☐ Another health care provider, please specify: ___________________

☐ I have shared my results with another individual. Please specify: ____________

B. If you shared your results with one or more of the health care providers below, choose the best option that describes your interaction with this provider. Please select only one response per row. If you did not share your results with any health care providers, proceed to question 4.

	**I did not share my results with this health care provider.**	**I informed him/her of the results, without further discussion.**	**I informed him/her of the results and had further discussion.**
**Primary care doctor**	o	o	o
**Parkinson's disease doctor** (other than the one who gave me my results)	o	o	o
**Genetic counselor** (other than the one who gave me my results)	o	o	o
**Mental health professional**	o	o	o
**Another health care provider;** please specify: ____________	o	o	o

C. If you discussed your results with one or more of the health care providers below, choose one or more of the options that describe your discussion with this provider. If you did not discuss your results with any of these health care providers below, proceed to question 4.

	**We discussed what these test results mean for me.**	**We discussed what these test results mean for my family.**	**This provider offered me emotional support.**	**We discussed genetic information (eg how a gene works, what a variant is, etc) related to my test results.**	**We discussed another topic related to my results.**
**Primary care doctor**	o	o	o	o	o
**Parkinson's disease doctor** (other than the one who gave me my results)	o	o	o	o	o
**Genetic counselor** (other than the one who gave me my results)	o	o	o	o	o
**Mental health professional**	o	o	o	o	o
**Another health care provider** (please specify): ______________	o	o	o	o	o

D. If you discussed another topic related to your results with one of more of these health care providers, please write in the table below the provider and the topic you discussed.

Health care provider	Topic discussed

4. We are interested in learning about medical and life changes that have occurred <u>since you received your genetic test results</u>. Please tell us whether each of these possible events has occurred, or not, and if it has, whether you think it is related to the gene test results.

	This has not happened since I received gene test results	This has happened, but I believe it is not related to the gene test results	This has happened, and I believe it is related to the gene test results
I have left a job.	o	o	o
I have lost a job.	o	o	o
I have started a new job.	o	o	o
I have had a change in a significant relationship (marriage, break-up, etc.).	o	o	o
I have started a new medication.	o	o	o
I have had surgery related to Parkinson's.	o	o	o
I discussed advanced care planning or advanced directives with my physician and/or family.	o	o	o
I have become interested in enrolling in a Parkinson's disease research study	o	o	o
*I have enrolled or I am in the process of enrolling in a Parkinson's disease research study.	o	o	o
I have moved to a new residence.	o	o	o

*If you have enrolled or are in the processing of enrolling in a Parkinson's disease research study, please share the name of the PD research study you have joined. If you do not recall the name, please describe the study:

____

____

5. Please write about any additional medical and life changes that have occurred since you received your genetic testing results.

____

____

____

6. Please indicate how useful you find your genetic test results regarding the following outcomes by checking the appropriate response. Please select one option for each row:

	Not at all useful	Somewhat useful	Neutral	Useful	Extremely Useful
Help with life planning	O	O	O	O	O
Inform my plans for school or career	O	O	O	O	O
Inform my decisions about having children (if relevant)	O	O	O	O	O
Help me or my family mentally prepare for the future	O	O	O	O	O
Help to better understand my health	O	O	O	O	O
Contribute to my self-knowledge	O	O	O	O	O
Help me cope with my health risks	O	O	O	O	O
Help me feel more in control of my health	O	O	O	O	O
Help me feel more in control of my life	O	O	O	O	O
Simply to provide information	O	O	O	O	O
Satisfy my curiosity	O	O	O	O	O
Help me to use social programs, like resources and services	O	O	O	O	O
Not at all useful	Somewhat useful	Neutral	Useful	Extremely Useful	
Improve communication with my family members	O	O	O	O	O
Feel good about helping the medical community	O	O	O	O	O

Feel good about having information for my family members	o	o	o	o	o
Feel good about taking responsibility for my children's health	o	o	o	o	o

7. As part of participating in this study, you received your test results in person (through your physician or a genetic counselor at the study site) or by phone (through a genetic counselor at Indiana University). How satisfied were you with receiving your genetic test results this way?
   - ☐ Very Satisfied
   - ☐ Satisfied
   - ☐ Neutral
   - ☐ Unsatisfied
   - ☐ Very Unsatisfied

   If applicable, please add any comments about your satisfaction with how you received your test results:
   ____
   ____
   ____

8. Would you have preferred to receive your genetic test results in a different way? Please select from below:
   - ☐ Yes
   - ☐ No

   A. If yes, which of the following ways would you have preferred to receive your genetic test results? (select the *one* most preferred option):
      - ☐ In person
      - ☐ Over the phone
      - ☐ Over the phone but with video so that both parties can see each other
      - ☐ By mail
      - ☐ By email
      - ☐ Other, please specify: ____________________________________

Supplementary Tables and Data

Supplementary Table 1: Participant demographics

	Total Participants (n=972)	Participants with negative genetic testing (n=835)	Participants with positive genetic testing (n=137)	P value
**Mean age in years (SD)**	64.7 (9.6)	65.2 (9.2)	61.7 (11.3)	$4.4x10^{-4}$
**Mean age of PD onset in years (SD)**	59.4 (10.4)	59.9 (10.0)	56.2 (12.0)	$3.2x10^{-4}$
**Early-onset (< 50 years) PD (%)**	161/972 (17%)	125/835 (15%)	36/137 (26%)	0.002
**First-degree relative with PD (%)**	222/824 (27%)	181/713 (25%)	41/111 (37%)	0.015
**Mean MoCA score (SD)**	26.7 (2.7)	26.7 (2.7)	26.7 (2.9)	0.729
**Gender (%)**				0.115
Female	421/972 (43%)	353/835 (42%)	68/137 (50%)	
**Race (%)**				0.603
African American	8/972 (1%)	8/835 (1%)	0/137 (0%)	
Asian & Pacific Islanders	18/972 (2%)	17/835 (2%)	1/137 (1%)	
White	924/972 (96%)	789/835 (96%)	135/137 (99%)	
Other	13/972 (1%)	12/835 (1%)	1/137 (1%)	
**Ethnicity (%)**				1.000
Hispanic	44/972 (5%)	38/835 (5%)	6/137 (4%)	
**High-risk ancestry[a] (%)**	164/972 (17%)	124/835 (15%)	40/137 (29%)	$1.1x10^{-4}$[b]
**Education (%)**				0.460
High school degree or fewer years	45/972 (5%)	36/835 (4%)	9/137 (7%)	
Bachelors degree or some college	459/972 (47%)	394/835 (47%)	65/137 (47%)	
Graduate degree	468/972 (48%)	405/835 (49%)	63/137 (46%)	
**Marital Status (%)**				0.595
Single or never married	89/972 (9%)	74/835 (9%)	15/137 (11%)	
Married/domestic partnership	765/972 (79%)	657/835 (79%)	108/137 (79%)	
Widowed, divorced, or separated	118/972 (12%)	104/835 (12%)	14/137 (10%)	
**Employment Status (%)**				0.202
Employed	320/972 (33%)	268/835 (32%)	52/137 (38%)	
Not employed	652/972 (67%)	567/835 (68%)	85/137 (62%)	
**Living Situation (%)**				0.320
Own home or with family	959/972 (99%)	825/835 (99%)	134/137 (98%)	
Assisted living facility or nursing home	7/972 (1%)	5/835 (1%)	2/137 (1%)	
Other	6/972 (1%)	5/835 (1%)	1/137 (1%)	

MoCA, Montreal Cognitive Assessment; PD, Parkinson's disease; SD, standard deviation

[a]High-risk ancestry includes Ashkenazi Jewish, Spanish Basque, and North African Berber
[b]Statistically significance difference between participants with negative genetic test results and participants with positive genetic test results

Supplementary Table 2: Summary of genetic testing results

**Genetic Testing Result**	**Number of Participants n=972**
Negative	835 (86%)
Positive	137 (14%)
GBA1	78 (8%)
p.N370S variant[b]	19 (2%)
p.E326K variant[b]	29 (3%)
p.L444P variant[b]	10 (1%)
Compound heterozygote or homozygote	2 (<1%)[a]
Other *GBA1* variant[b]	18 (2%)
LRRK2	25 (3%)
p.G2019S variant[b]	20 (2%)
Other *LRRK2* variant[b]	5 (<1%)
PRKN	22 (2%)
Heterozygote	12 (1%)
Compound heterozygote or homozygote	10 (1%)
SNCA	3 (<1%)
PARK7	2 (<1%)
PINK1	1 (<1%)
More than one gene	6 (<1%)

[a]One individual is homozygous for p.N370S. One individual is compound heterozygous with an p.L444P variant and another *GBA1* variant. No *VPS35* variants were identified in this population.
[b]Heterozygous individual

Supplementary Table 3: Participant-reported personal utility of genetic test results by age

Survey Item	Mean (SD) Age Endorsed as Useful	Mean (SD) Age Endorsed as Not Useful	P value
**Satisfy my curiosity**	64.6 (9.9)	65.3 (8.7)	0.610
**Feel good about helping the medical community**	64.6 (9.7)	62.3 (10.1)	0.077
**Simply to provide information**	64.4 (9.9)	63.8 (9.5)	0.643
**Feel good about having information for my family members**	64.5 (9.8)	64.7 (9.1)	0.897
**Contribute to my self-knowledge**	64.1 (9.8)	65.0 (10.3)	0.525
**Feel good about taking responsibility for my children's health**	64.1 (10.0)	65.6 (8.7)	0.034
**Help to better understand my health**	63.6 (9.9)	66.3 (9.2)	0.001
**Help me cope with my health risks**	63.3 (10.0)	66.0 (8.9)	1.0×10^{-4}[a]
**Help me feel more in control of my health**	63.2 (9.9)	65.5 (9.3)	0.002
**Improve communication with my family members**	63.9 (10.0)	64.8 (9.4)	0.229
**Help me feel more in control of my life**	63.5 (10.0)	65.5 (9.2)	0.004
**Help me to use social programs, like resources and services**	63.7 (10.2)	65.5 (9.4)	0.010
**Help with life planning**	64.1 (10.4)	65.5 (9.5)	0.058
**Help me or my family mentally prepare for the future**	63.7 (10.5)	66.0 (9.0)	0.002
**Inform my plans for school or career**	61.2 (10.6)	66.0 (9.1)	2.3×10^{-8}[a]
**Inform my decisions about having children**	60.6 (11.2)	65.8 (9.0)	6.8×10^{-8}[a]

[a]Statistically significance difference in age between participants who endorsed an item as useful and participants who endorsed an item as not useful

Supplementary Table 4: Participant-reported impact of genetic test results

Category	Response
Lifestyle changes (n=16 responses)	"I've recommitted to consistent, frequent, vigorous exercise. I've reevaluated my diet." -Gene negative participant
	"I am moving into semi-retirement because of my disease but not due to genetic testing" -Gene negative participant
	"Exercised more and will continue to do so. Watching my blood pressure more due to higher highs and lower lows. Rest more. I try to take much better care of myself." -Gene positive participant
Positive emotions (relief, gratefulness, happiness) (n=8 responses)	"I am more at ease and less anxious regarding the possible hereditary aspect of my Parkinson's. I do understand that that the gene study is still ongoing, and the hereditary aspect is not 100%." -Gene negative participant
	"The results say to expect slow progression. I know that this is no guarantee, but it has relaxed me some." -Gene positive participant
Informed family members (n=4 responses)	"I have a daughter who has probable young onset Parkinson's disease. She has 6 children. When I was given a negative result on the initial report, I called to let her know. We were both relieved." -Gene negative participant
	"Talked more with my adult children about results & implications for them." -Gene positive participant
Negative emotions (concern, anxiety) (n=2 responses)	"Concerned about the how the genetic results affect with some of the family, regarding taking family trips or doing activities as a family. Concern health needs will affect the rest of my family member" -Gene positive participant
	"I have become more worried that I might have contracted another life-threatening disease that is not Parkinson's. My mortality is more on my mind." -Gene positive participant

Supplementary Table 5: Participant-reported personal utility of genetic test results by genetic subgroup

Survey Item	Negative results (n=835)<br><br>% endorsed as useful	Positive results (n=137)<br><br>% endorsed as useful	*GBA1* p.E326K (n=29)<br><br>% endorsed as useful	*GBA1* LP/P variants (n=47)<br><br>% endorsed as useful	*LRRK2* (n=25)<br><br>% endorsed as useful	*PRKN,* cp het/homo (n=10)<br><br>% endorsed as useful	*PRKN* het (n=12)<br><br>% endorsed as useful
**Satisfy my curiosity**	80% (659/822)	85% (115/135)	90% (26/29)	72% (34/47)	88% (21/24)	100% (9/9)	92% (11/12)
**Feel good about helping the medical community**	80% (658/820)	82% (112/137)	86% (25/29)	74% (35/47)	88% (22/25)	90% (9/10)	83% (10/12)
**Simply to provide information**	77% (631/818)	85% (116/137)	90% (26/29)	79% (37/47)	76% (19/25)	90% (9/10)	92% (11/12)
**Feel good about having information for my family members**	76% (627/821)	78% (107/137)	72% (21/29)	74% (35/47)	84% (21/25)	80% (8/10)	75% (9/12)
**Contribute to my self-knowledge**	68% (558/822)	82% (113/137)	83% (24/29)	79% (37/47)	80% (20/25)	100% (10/10)	83% (10/12)
**Feel good about taking responsibility for my children's health**	63% (517/816)	62% (84/136)	62% (18/29)	64% (30/47)	42% (10/24)	80% (8/10)	58% (7/12)
**Help to better understand my health**	50% (408/822)	62% (85/137)	69% (20/29)	55% (26/47)	60% (15/25)	80% (8/10)	50% (6/12)
**Help me cope with my health risks**	48% (392/822)	56% (76/136)	52% (15/29)	64% (30/47)	52% (13/25)	70% (7/10)	36% (4/11)
**Help me feel more in control of my health**	48% (396/822)	52% (71/136)	52% (15/29)	55% (26/47)	44% (11/25)	80% (8/10)	36% (4/11)
**Improve communication with my family members**	47% (385/820)	51% (70/137)	52% (15/29)	55% (26/47)	44% (11/25)	80% (8/10)	33% (4/12)
**Help me feel more in control of my life**	46% (379/820)	54% (73/136)	59% (17/29)	55% (26/47)	48% (12/25)	80% (8/10)	36% (4/11)
**Help me to use social programs, like resources and services**	39% (319/820)	46% 63/137	55% (16/29)	47% (22/47)	40% (10/25)	40% (4/10)	42% (5/12)
**Help with life planning**	38% (313/821)	47% (64/137)	52% (15/29)	43% (20/47)	40% (10/25)	60% (6/10)	42% (5/12)
**Help me or my family mentally prepare for the future**	36% (298/823)	44% (60/137)	41% (12/29)	47% (22/47)	28% (7/25)	70% (7/10)	42% (5/12)
**Inform my plans for school or career**	20% (168/821)	25% (34/136)	29% (8/28)	28% (13/47)	20% (5/25)	40% (4/10)	17% (2/12)

**Inform my decisions about having children**	17% (139/811)	21% (29/135)	25% (7/28)	22% (10/46)	16% (4/25)	30% (3/10)	25% (3/12)

Supplementary Table 6: Research interest and participation by genetic subgroup

Survey item	Negative results (n=835)	Positive results (n=137)	*GBA1* p.E326K (n=29)	*GBA1* LP/P variants (n=47)	*LRRK2* (n=25)	*PRKN*, cp het/homo (n=10)	*PRKN* het (n=12)
**I have become interested in enrolling in a Parkinson's disease research study** (due to gene test results).	11% (94/835)	33% (45/137)	24% (7/29)	36% (17/47)	24% (6/25)	30% (3/10)	17% (2/12)
**I have enrolled or I am in the process of enrolling in a Parkinson's disease research study** (due to gene test results).	3% (26/835)	10% (14/137)	7% (2/29)	6% (3/47)	4% (1/25)	20% (2/10)	8% (1/12)

Supplementary Data (excel file): Participant-reported personal utility of genetic test results by demographic variables
